# Transcription Factor MYB as Therapeutic Target: Current Developments

**DOI:** 10.3390/ijms25063231

**Published:** 2024-03-12

**Authors:** Karl-Heinz Klempnauer

**Affiliations:** Institute for Biochemistry, University of Münster, 48149 Münster, Germany; klempna@uni-muenster.de

**Keywords:** MYB, inhibitor, AML, leukemia, adenoid cystic carcinoma, transcription, p300, gene expression

## Abstract

The MYB protein is a pivotal player in the cellular transcriptional network, influencing major important processes such as cell proliferation, differentiation, and apoptosis. Because of its role in oncogenesis, MYB is now a compelling target for therapeutic interventions in cancer research. This review summarizes its molecular functions and current therapeutic approaches aiming to inhibit its oncogenic activity.

## 1. The Discovery of the *MYB* Gene

Transcription factors pose a unique challenge for pharmacological targeting due to their lack of well-defined binding sites, unlike enzymes with distinct cavities. The intricate functions of transcription factors, primarily their interaction with DNA and other proteins, involve extensive surface areas that are generally unsuitable for small-molecule inhibitors. However, recent advances highlight MYB as a promising exception in this context. Substantial strides have been taken in unraveling the pivotal role of MYB in human leukemia and various cancers. Moreover, pioneering pilot studies have successfully demonstrated the feasibility of pharmacologically inhibiting MYB. This review provides a comprehensive overview of the current landscape surrounding MYB as an emerging and promising therapeutic target.

**Figure 1:**
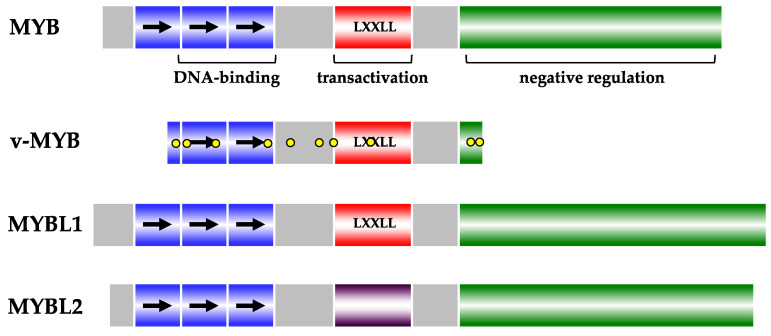
The vertebrate MYB family. Functional domains for DNA-binding, transactivation and negative regulation are highlighted. The repeat structure of the DNA-binding domain is depicted by the arrows. Amino acid replacements in v-MYB are indicated by yellow dots. The LXXLL motif facilitating interaction with the KIX domain of p300 is shared by MYB, v-MYB and MYBL1. MYBL2 possesses an unrelated transactivation domain. Refer to the text for additional details.

*MYB* is the founding member of the *MYB* family, a conserved group of genes of vertebrate animals and humans. In vertebrates, the *MYB* family consists of *MYB* itself and the *MYB*-related genes *MYBL1* and *MYBL2* (Figure 1). *MYB*-related genes are also found in the genomes of many non-vertebrate animal species and plants. In plants, they form a particularly large and diverse gene family with crucial roles in developmental processes, metabolism, and response to environmental stimuli [1,2,3].

*MYB* was initially discovered in studies of the avian myeloblastosis virus (AMV), an oncogenic chicken retrovirus causing myelomonocytic leukemia in chickens. AMV belongs to a larger group of acutely transforming retroviruses that infect different animal species and owe their oncogenic potential to the presence of specific nucleotide sequences transduced from the genomes of their respective hosts. In the case of AMV, the oncogenic sequence was designated as viral *myb* gene (v-*myb*), while its cellular counterpart, initially named c-*myb*, is now referred to as *MYB*. Notably, the retrovirally transduced gene is not an exact copy of its cellular counterpart, but differs from it by multiple changes, including point mutations and the N- and C-terminal truncations of the protein coding sequence. These changes contribute to the oncogenic potential of AMV [4,5].

In vertebrate organisms, *MYB* is highly expressed mainly in immature hematopoietic cells. *MYB* is essential for the development of the hematopoietic system: *MYB*-deficient mice die during embryonic development due to multiple defects affecting most hematopoietic lineages [6]. Lower levels of *MYB* expression occur in the stem cells of the intestinal crypts, in breast cells and in certain neural cell populations [7].

## 2. The MYB Protein

The proteins encoded by the viral and cellular *MYB* genes, designated as v-MYB and MYB, serve as nuclear DNA-binding proteins, functioning as bona fide transcription factors [8,9,10]. The N-terminal part of MYB comprises three or two (in case of the viral protein) imperfect copies of a 50-amino-acid stretch each, forming the DNA-binding domain. This part of MYB is reminiscent of helix–turn–helix (HTH) type DNA-binding domains; however, unlike classical HTH-proteins, MYB recognizes specific DNA sequences as monomers. This MYB DNA binding domain’s basic structure is conserved not only among other vertebrate MYB-family members but also among non-vertebrate and plant MYB-type proteins. Alongside the DNA-binding domain, another pivotal element of MYB is the transactivation domain, located in the central part of the MYB amino acid sequence. Lastly, the C-terminal part of MYB, termed the negative regulatory domain, is absent in the virally transduced oncogenic version, and the artificial C-terminal truncation of MYB contributes to the activation of its oncogenic potential. This part of MYB is influenced by signaling pathways and is believed to exert its regulatory function by folding back on the DNA-binding domain [8,9,10].

The role of MYB as a transcription factor was initially suspected due to its sequence-specific DNA-binding activity and transactivation potential in reporter assays [11,12]. This suspicion was eventually confirmed with the identification of the chicken *mim*-1 gene as a direct MYB target gene [13]. Subsequent work, utilizing genome-wide techniques, unveiled a diverse array of genes regulated by MYB in hematopoietic or other cells [14,15,16,17]. Consistent with this, genome-wide chromatin immunoprecipitation (ChIP) and genomic footprinting studies in human leukemia cells exposed numerous binding sites in the promoters and other regulatory regions of its target genes [17,18,19,20]. Genome-wide ChIP studies also demonstrated MYB’s colocalization with other key transcription factors crucial in hematopoietic cells, emphasizing the cooperative nature of target gene regulation by MYB [18,21].

**Figure 2:**
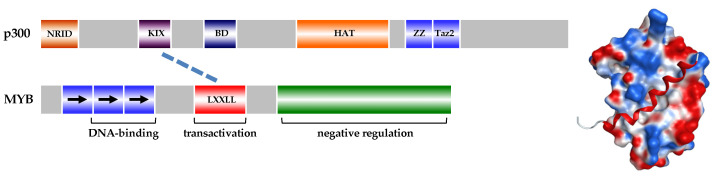
The left part of the figure schematically illustrates the domain structure of p300 and highlights the interaction between the LXXLL-motif of MYB and the KIX domain of p300. Other p300 domains shown: NRID, nuclear receptor interaction domain; BD: bromodomain; HAT, histone acetyltransferase domain; ZZ, ZZ domain; Taz2, Taz2 domain [24,25]. The right part of the figure shows the docking of a helical peptide containing the LXXLL-motif (red) to a groove on the surface of the KIX domain. The electrostatic potential of surface areas is indicated by blue (positive) and red (negative) colors. The right part of the figure is reproduced from [33].

In various studies exploring protein interactions with MYB [8], the coactivator p300/CBP emerged as a pivotal MYB interaction partner [22,23]. p300 and the related CREB binding protein (CBP) function as acetyl transferases, modifying lysine residues in histones and in other proteins [24,25]. Beyond their enzymatic activity, p300 and CBP present multiple surfaces for interaction with various transcription factors, allowing them to be recruited to their genomic sites of action [24,25]. The binding of p300/CBP to MYB is facilitated by a conserved LXXLL amino acid motif in the MYB transactivation domain, interacting with the KIX domain of the coactivator (Figure 2) [26]. Despite their overall similarity, p300 and CBP play distinct roles in hematopoietic cells [27]. The interaction with p300 is crucial for the intrinsic transforming capacity of MYB and its pro-leukemogenic role in vitro [28] and in vivo. This was demonstrated by utilizing mutant alleles of MYB or p300 that disrupt their interaction, preventing leukemia formation in mice [29]. Due to p300’s multiple surfaces serving as docking sites for various proteins, p300 can orchestrate the formation of MYB-containing transcriptional complexes with cooperating transcription factors. An example is the p300-mediated cooperation of MYB with the CCAAT-box enhancer binding protein beta (C/EBPβ), which binds to the Taz2 domain of p300 [30]. The cooperation of MYB and C/EBPβ is critical for myeloid-specific gene expression [31,32].

## 3. The Role of MYB in Human Cancer

**MYB and leukemia.** Because v-*MYB* was initially identified as the oncogene of a leukemia-inducing chicken retrovirus, it was not surprising that many human leukemia cell-lines show significant *MYB* expression [7]. However, whether *MYB* actively contributed to leukemia development in humans or if its expression in leukemia cells merely reflected its normal expression in immature hematopoietic cells remained unclear. An active role for *MYB* in leukemia development was suggested by the discovery of the recurrent chromosomal alterations of the *MYB* gene in T-cell acute lymphoblastic leukemia (T-ALL) [34,35,36]. These alterations, including duplications and translocations, led to increased *MYB* expression in leukemia cells. A recurrent translocation was also detected in rare cases of acute basophilic leukemia in which *MYB* was fused to the *GATA1* gene, causing the expression of a MYB-GATA1 fusion protein [37,38]. Recent research has further supported MYB’s active role in leukemia development, demonstrating that genomic alterations can lead to the formation of de novo MYB binding sites in the vicinity of other oncogenes, stimulating their expression. This was initially detected in T-ALL where the generation of de novo MYB binding sites in the upstream region of the *TAL1* oncogene creates a MYB-dependent neo-enhancer driving the expression of *TAL1* [39]. Other studies identified genomic alterations creating a MYB-dependent promoter in an intron of the *LMO2* gene [40] or a MYB-dependent neo-enhancer downstream of *TAL1* [41]. Adding complexity to the idea that non-coding mutations can impact MYB binding sites and contribute to cancer, mutations in a cis-acting element in an intron of the *WT1* gene were found to destroy MYB binding and downregulate the tumor suppressor gene *WT1* in acute promyelocytic leukemia [42].

Unlike T-ALL, direct genomic rearrangements affecting MYB in acute myeloid leukemia (AML) are rare. However, MYB plays a pivotal role for the proliferation and survival of the AML cells transformed by other leukemia oncogenes [43,44,45]. AML cells are more dependent on MYB than normal hematopoietic progenitor cells, being described as addicted to high levels of MYB expression. This dependence makes AML cells more sensitive than normal hematopoietic progenitors to the down-modulation of MYB expression [46,47] and provides a rationale for considering MYB as a therapeutical target for AML. This idea was supported experimentally by mouse model studies. In these studies, mice were engrafted with hematopoietic progenitors transformed by a retroviral construct encoding an MLL-AF9 fusion protein, resulting in the development of an aggressive AML. It was observed that the downregulation of MYB expression eradicated the MLL-AF9 induced AML without affecting normal hematopoiesis simultaneously [45]. The concept that the partial inhibition of MYB could be a therapeutic strategy for AML has led to various studies aiming to identify compounds as pharmacological inhibitors of MYB.

**MYB and solid cancers.** MYB has also been implicated in the development of certain non-hematopoietic malignancies. Recurrent translocations between *MYB* and *NFIB* occur in a high percentage of cases of adenoid cystic carcinoma (ACC), resulting in the expression of oncogenic MYB/NFIB fusion proteins [48,49]. More recently, translocations of *NFIB* with another *MYB* family member, *MYBL1*, have also been described [50]. The MYB DNA-binding and transactivation domains are usually retained in the fusion proteins whereas the C-terminal part of MYB is truncated to different extent, depending on the exact position of the translocation breakpoint within the *MYB* gene. Additionally, the fusion proteins are strongly overexpressed in the ACC cells [48,49]. It has been proposed that the translocation uncouples the expression of the fusion proteins form inhibitory mechanisms mediated by micro-RNAs acting on sequences in the 3′ untranslated part of the *MYB* mRNA. Furthermore, the increased expression of the MYB fusion proteins in ACC cells may also result from the juxtaposition to *NFIB* enhancer elements or copy number gains [51,52]. In analogy to the discovery of *MYB*/*NFIB* fusions in ACC cells, recent work has also uncovered recurrent translocations of *MYB* or *MYBL1* with several other genes in pediatric low-grade gliomas [53,54,55].

The deregulation of *MYB* has also been implicated in solid cancers of the breast, the colon, the pancreas and the prostate. In breast cancer, a high percentage of estrogen receptor-positive tumors show high MYB expression [56]. In vitro and in vivo studies have demonstrated that MYB stimulates proliferation and tumor growth and suppresses apoptosis [56,57,58,59,60]. In colon cancer, increased levels of *MYB* expression also correlate with poor prognosis [61]. Mechanistically, mutations in the intron 1 of *MYB* have been proposed to cause increased expression in colon cancer cells by mediating a more efficient read-through in the transcriptional attenuator region in the intron [61,62,63,64]. In pancreas cancer, *MYB* was initially identified as a candidate oncogene due to its amplification in approximately 10% of cancers cases [65]. MYB is generally highly expressed in most pancreatic cancer cells, irrespective of gene amplification, and is responsible for promoting their growth and malignant properties [66]. Recent work has identified the role of MYB in the hypoxic survival of the cancer cells, mediated by its ability to stimulate *HIF1a* expression and function [67]. In prostate cancer, *MYB* was also initially found to be amplified, especially in hormone-refractory cancer cases [68]. In these cells, MYB expression overrides cell cycle arrest and apoptosis induced androgen depletion and confers aggressive malignant properties [69]. Recently, MYB has been shown to interact with the androgen receptor and to sustain its ligand-independent activation [70]. Overall, these findings support the notion that MYB can also act as an oncogenic “driver” in certain non-hematopoietic tumors, making it an intriguing therapeutic target for these malignancies.

## 4. MYB as a Therapeutic Target

**Inhibition of the MYB transactivation function**. Directly inhibiting the transactivation function of MYB has garnered increased attention as a potential therapeutic strategy, given recent insights into its role in leukemia and certain non-hematopoietic malignancies. Previous work focused on diminishing *MYB* expression using nucleic acid-based antisense or ribozyme strategies [46,47,71,72]. However, more recent studies have explored the targeting of MYB’s transcriptional activity. The stimulation of target genes by MYB strongly depends on its association with co-activators p300 and the CREB-binding protein (CBP). These coactivators bind to the LXXLL amino acid sequence motif in the MYB transactivation domain via their KIX domain (Figure 2) [26]. Notably, mutations affecting the LXXLL motif or KIX domain result in decreased MYB activity by weakening the interaction of both proteins, causing various hematopoietic defects in mice [73,74,75]. Pattabiraman et al. showed that the interaction of MYB and p300 is crucial for the proliferation and survival of AML cells [29]. Consequently, disrupting the MYB-p300 interaction emerged as a pertinent strategy for targeting MYB activity in leukemia. Previous studies identified Naphthol-AS E phosphate, a chemical compound capable of binding to the KIX domain of CBP, which suppressed its interaction with the cyclic AMP response element binding protein (CREB) [76]. By using a bacterial autodisplay expression system to monitor the MYB-KIX interaction, it was demonstrated that Naphthol-AS E phosphate inhibited the interaction between MYB and p300, thereby suppressing the transcriptional activity of MYB on one of its endogenous target genes [77]. This study provided proof-of-principle that targeting of MYB transcriptional activity with a chemical compound is feasible and showed that the inhibition of the MYB-p300 interaction induces differentiation and apoptosis in human myeloid leukemia cell lines. Furthermore, these findings suggested that the disruption of the MYB-p300 interaction might be a valid strategy for the treatment of leukemia. A subsequent study revealed that the natural compound Celastrol exhibited a significantly higher affinity to the KIX domain than Naphthol-AS E phosphate and, more potently, inhibited the MYB-KIX interaction [33]. Celastrol, in addition to inhibiting MYB’s transcriptional activity, reduced the proliferative capacity of AML cells, induced their differentiation and apoptosis and delayed the development of leukemia in an in vivo mouse model of AML [33]. Importantly, colony formation assays in semi-solid medium showed that leukemic cells from AML patients were more sensitive to Celastrol than normal hematopoietic progenitor cells, which is consistent with previous findings that highlight the addiction of AML cells to MYB. Another study employing the bacterial autodisplay assay of the MYB-KIX interaction identified several naphthoquinones, including Plumbagin, that interfered with the MYB-KIX interaction [78]. Plumbagin, similar to Celastrol, suppressed the expression of direct MYB target genes and induced myeloid differentiation and apoptosis. As in case of Celastrol, Plumbagin inhibited the proliferation of patient-derived AML to a stronger extent than the proliferation of normal hematopoietic progenitors, which is in line with the MYB addiction of the leukemia cells. Unlike Celastrol, however, these naphthoquinones were shown to primarily bind to the Myb transactivation domain rather than to the KIX domain [78].

The Kentsis group pursued an alternative approach to develop a MYB-p300 inhibitor, utilizing a peptide-based mimic of the LXXLL motif within the MYB transactivation domain. The peptide mimic disrupted the MYB-p300 interaction by binding to the KIX domain, effectively suppressing the MYB-dependent transcriptional program in AML cells. Moreover, in vitro studies demonstrated that the mimic induced effects similar to Celastrol in AML cells, while in vivo experiments showed the prolonged survival of mice engrafted with patient-derived AML cells, mirroring the effects observed with Celastrol [21,79]. Encouraged by these promising results, various laboratories are currently engaged in the development of additional MYB-p300 inhibitory compounds [80,81,82,83].

The peptidomimetic inhibition of MYB-dependent gene expression in AML cells has provided further insights into MYB’s cooperation with various transcription factors in a p300/CBP-dependent manner [21]. Co-localization studies revealed that numerous transcription factors accompany MYB and p300 at multiple regulatory regions in AML cell chromatin [18]. These complexes likely contribute to the MYB-dependent transcriptional program in AML cells, offering potential avenues for novel therapeutic interventions. Notably, the cooperation of MYB and p300 with members of the CCAAT-box enhancer binding protein (C/EBP) family has been well-established in myeloid-specific gene expression, with recent research showing that certain C/EBPβ inhibitory compounds indirectly suppress the MYB-dependent transcriptional program in AML cells [30,31,32]. These compounds, including natural sesquiterpene lactones and synthetic mimics, demonstrate potent antiproliferative- and differentiation- or apoptosis-inducing properties on AML cells in vitro. Strikingly, these compounds exert a significantly stronger suppression of proliferation in leukemic cells from AML patients compared to normal myeloid progenitors, underscoring the MYB addiction of AML cells and highlighting the relevance of C/EBPβ as a MYB-p300 cooperation partner for the maintenance of AML cells [84,85].

Studies from the Vakoc laboratory have uncovered a novel dependency of AML cells on TAF12, a subunit of the TFIID and SAGA coactivator complex that links MYB to the basal transcriptional machinery [86]. The authors observed that AML cells, but not normal myeloid progenitors, have a specific requirement for TAF12 expression. Further work revealed that a TAF12/TAF4 heterodimer interacts with the MYB transactivation domain. Intriguingly, the expression of a TAF4 histone-fold fragment squelched TAF12 and thereby suppressed MYB oncogenic activity, both in AML cells in vitro and in an in vivo mouse model of AML. These findings raise interesting questions regarding the details of the TAF12/TAF4-MYB interaction in relation to the MYB-p300 interaction and suggest a novel strategy for targeting MYB in AML.

**Employing cell-based screening approaches to identify MYB inhibitors.** Several groups have employed cell-based assays to screen chemical compound libraries for MYB inhibitory compounds. Yusenko et al. [87] utilized human embryo kidney (HEK) cells equipped with an inducible MYB expression system and a luciferase read-out as screening tool. This identified several compounds, including teniposide (a topoisomerase inhibitor), monensin (a polyether ionophore), oprozomib (a proteasome inhibitor), LAQ824 (a HDAC-inhibitor), several inhibitors of tyrosine protein kinases and a novel chemical compound (Bcr-TMP) as potential MYB-inhibitors [87,88,89,90,91,92]. All these compounds suppressed MYB transcriptional activity and downregulated direct MYB target genes. Further analysis revealed that these compounds suppressed MYB activity by disturbing its cooperation with p300. However, unlike compounds that interfere with the MYB-KIX interaction, these compounds suppressed the cooperation of MYB and p300 probably indirectly by still unknown mechanisms. All these inhibitory compounds induced differentiation and apoptosis of AML cells and suppressed their proliferation to a significantly stronger extent than the proliferation of non-hematopoietic cells. Notably, these effects were MYB-dependent, i.e., they were counteracted by the expression of a truncated (i.e., activated) version of MYB. In vitro assays showed that primary AML cells from mice or from human AML patients were more sensitive to some of the compounds than normal hematopoietic progenitors. It is also interesting that some of the compounds, when assessed for their effects on patient-derived ACC cells, were found to suppress the proliferation of these cells more strongly than the proliferation of control cells not expressing MYB-NFIB fusion proteins.

Mandelbaum et al. [93] utilized zebrafish blastomeres engineered to express an endogenous MYB-GFP fusion protein as a cell-based system to screen a chemical library for compounds that suppress GFP fluorescence by inhibiting MYB-GFP expression. They identified all-trans retinoic acid (ATRA) as an inhibitor of endogenous MYB expression, showing that it downregulates the expression of the MYB-NFIB fusion protein in ACC cells in vitro and suppresses tumor growth in ACC patient-derived xenograft models in vivo. Mechanistically, the ATRA-mediated downregulation of MYB-NFIB expression decreased its binding at translocated enhancers, thereby diminishing a feedback loop driving ACC. A first phase II clinical trial on the effects of ATRA in advanced adenoid cystic carcinoma is already ongoing [94].

Walf-Vorderwülbecke et al. [95] employed a drug repurposing strategy, using the Connectivity Map (CMAP) database to compare drug-specific gene expression profiles with the AML-specific gene expression signature. This led to the identification of the anti-helminth drug mebendazole as potential MYB inhibitor. Mebendazole suppressed the proliferation of several AML cell lines by inducing the proteasomal degradation of MYB, thereby blocking its transcriptional program. Furthermore, colony formation by leukemia cells from two AML patients was blocked more efficiently than the proliferation of normal hematopoietic progenitors and the onset of leukemia in a mouse model of AML was delayed by mebendazole. The same strategy was employed by the authors to identify another MYB inhibitory drug, withaferin A (WFA) [96]. Like mebendazole, WFA suppressed the proliferation of AML cells by the ablation of MYB expression due to the induction of an unfolded protein response. Leukemia cells from AML patients were inhibited to a significantly stronger extent than normal CD34-positive normal hematopoietic progenitor cells. Finally, WFA was found to impair AML progression in a mouse model. Interestingly, WFA had previously been identified as a potent inhibitor of C/EBPβ, suggesting that its effect in AML cells might also be due to indirect inhibition of MYB in a MYB-p300-C/EBPβ context [97].

Tejera Nevado et al. [98] recently employed two T-ALL cell lines (Molt-4 and CCRF-CEM) and a qPCR read-out for *MYB* mRNA expression to screen an inhibitor library for compounds that downregulate *MYB* expression. This strategy identified two related oleanane triterpenoids, bardoxolone methyl and omaveloxolone, potently suppressing *MYB* expression in both cell lines. Both compounds reversed the MYB-driven transcriptional program and induced apoptosis in T-ALL cells. Interestingly, the compounds synergized with doxorubicin, a DNA-damaging agent that is part of the standard therapy for T-ALL. Since both compounds are in late clinical stage development or have already been approved for other diseases, it will be interesting to see if they can be repurposed for T-ALL and other MYB-driven leukemias. Notably, bardoxolone methyl, which is also known as methyl ester of 2-Cyano3,12-dioxooleana-1,9-dien-28-oic acid (CDDO-Me), has already been described for its effects on AML cells, inducing strong antiproliferative effects, apoptosis and differentiation in AML cell lines and primary AML samples [99,100]. In colony formation assays, the inhibitory effects of the compound were significantly stronger on primary AML and CML cells than on normal CD34-positive hematopoietic progenitors. CDDO-Me is a multifunctional compound with a broad range of applications, probably due to its ability to undergo covalent addition reactions with cysteine residues [101]. The molecular mechanism of how CDDO-Me and omaveloxolone inhibit MYB expression is not clear. It is interesting that these compounds share structural similarity with Celastrol, which disrupts the MYB-p300 interaction by binding to the KIX domain [33]. Thus, it would be interesting to investigate whether the oleane triterpenoids share this activity with Celastrol. In any case, the differential effects of CDDO-Me on leukemic and normal hematopoietic progenitor cells suggest that CDDO-Me and omaveloxolone may have potential for the treatment of hematologic malignancies.

The Sala and Stenman groups recently exploited critical downstream MYB targets to disrupt MYB oncogenic activity in ACC cells by interfering with downstream events. Using the ectopic expression of full-length MYB or a MYB-NFIB fusion protein in the non-tumorigenic MCF10A beast epithelial cell line they created a simplified model of ACC [102]. The resulting cells showed characteristics of transformation, such as increased proliferation and formation of organoids in three-dimensional culture. Utilizing this cell model the DNA-damage sensor protein kinase ATR was identified as a critical downstream target of MYB and MYB-NFIB. The ATR inhibitor VX-970 reversed the phenotypic changes induced by MYB or MYB-NFIB expression, suggesting that ATR is a critical effector in ACC. This was confirmed by investigating the effect of VX-970 on patient-derived ACC cell lines in vitro and on a PDX-mouse model of ACC in vivo [102]. The Sala group also generated a variant version of their MCF10A-based cell model, carrying a doxycycline-inducible instead of a constitutively expressed MYB transgene. They focused on the mitotic checkpoint kinase BUB1 as a direct MYB downstream target [103] and showed that the selective BUB1 inhibitor BAY1816032 caused the growth arrest and apoptosis of patient-derived ACC cells.

In conclusion, diverse approaches have been instrumental in identifying potential MYB inhibitors, offering promising avenues for therapeutic interventions in various cancers, particularly AML, T-ALL and ACC. These studies have revealed compounds targeting different aspects of MYB function, including the disruption of the MYB-p300 interaction, the induction of proteasomal degradation and interference with downstream effectors. The findings underscore the potential of repurposing existing drugs. Despite being in the early stages of research, the results hold promise for the development of novel therapeutics targeting MYB in hematologic and other malignancies.

## 5. Concluding Remarks

There is now compelling evidence to view MYB as a promising therapeutic target for AML and ACC, and for other malignancies driven by dysregulated MYB. Despite transcription factors traditionally being considered “undruggable”, recent research from various laboratories has pinpointed the MYB-p300 interaction as a vulnerable point for MYB. Recent studies have successfully demonstrated the feasibility of disrupting this interaction using small molecule inhibitors, proposing a viable strategy for treating MYB-driven tumors. Alongside the direct inhibition of the MYB-p300 interaction, numerous compounds inhibiting MYB oncogenic activity have been identified. These may act in indirect ways or induce the downregulation of MYB expression.

Despite notable progress, work on MYB inhibitors is still in its early stages, with virtually all studies at a pre-clinical level. Many key questions remain unresolved regarding the application of MYB inhibitors in human cancer patients and their potential side effects. Some of the compounds, such as Celastrol, the naphthoquinones or the oleanane triterpenoids, possess cysteine-reactive chemical groups. These compounds can lead to adverse side effects that may preclude their use in human patients. Another important issue is the long-term effect of MYB inhibition on the cancer cells. Targeted therapies often result in the cancer cells developing resistance by various mechanisms. These questions can be addressed as the work progresses to a clinical stage.

In summary, the work reviewed here has laid the foundation for developing strategies to inhibit MYB. Further research along these lines will deepen our comprehension of MYB’s function in human malignancies and, optimistically, yield compounds suitable for the therapeutic intervention in these cancers.
